# Can Hippocampal Neurites and Growth Cones Climb over Obstacles?

**DOI:** 10.1371/journal.pone.0073966

**Published:** 2013-09-06

**Authors:** Thuy Linh Lien, Jelena Ban, Massimo Tormen, Elisa Migliorini, Gianluca Grenci, Alessandro Pozzato, Vincent Torre

**Affiliations:** 1 Neurobiology Sector, International School for Advanced Studies (SISSA), Trieste, Italy; 2 Istituto Officina dei Materiali (IOM-CNR), Basovizza, Trieste, Italy; Rutgers University, United States of America

## Abstract

Guidance molecules, such as Sema3A or Netrin-1, can induce growth cone (GC) repulsion or attraction in the presence of a flat surface, but very little is known of the action of guidance molecules in the presence of obstacles. Therefore we combined chemical and mechanical cues by applying a steady Netrin-1 stream to the GCs of dissociated hippocampal neurons plated on polydimethylsiloxane (PDMS) surfaces patterned with lines 2 µm wide, with 4 µm period and with a height varying from 100 to 600 nm. GC turning experiments performed 24 hours after plating showed that filopodia crawl over these lines within minutes. These filopodia do not show staining for the adhesion marker Paxillin. GCs and neurites crawl over lines 100 nm high, but less frequently and on a longer time scale over lines higher than 300 nm; neurites never crawl over lines 600 nm high. When neurons are grown for 3 days over patterned surfaces, also neurites can cross lines 300 nm and 600 nm high, grow parallel to and on top of these lines and express Paxillin. Axons - selectively stained with SMI 312 – do not differ from dendrites in their ability to cross these lines. Our results show that highly motile structures such as filopodia climb over high obstacle in response to chemical cues, but larger neuronal structures are less prompt and require hours or days to climb similar obstacles.

## Introduction

The formation of the appropriate connections among neurons in all nervous systems requires developing neurons to find the correct target [1,2]. During this process, neurons navigate in the environment searching for guidance cues, leading to the appropriate connections. Growth cones (GCs) located at the tip of growing axons are the major motile structures guiding neuronal navigation [3–7]. GCs are composed of a lamellipodium, flat (sheet-like) protrusion from which thin finger-like projections called filopodia emerge [3,6,8] and are decorated by a variety of receptors able to sense the presence of appropriate chemical cues, such as guidance molecules. Filopodia exploratory motion is controlled and tuned by guidance molecules, which can attract or repel them.

Four main families of guidance molecules have been identified: Netrins, Slits, Semaphorins, and Ephrins [2,9–11]. The intracellular signaling mechanisms by which GCs convert these signals into directional decisions [12] are only partially understood. Membrane microdomains seem responsible for generating the localized signals producing specific responses to guidance cues. Calcium signaling and focal adhesion molecules control GC steering and modulate its response [1,13].

In addition to biochemical stimuli, several mechanical factors influence neuronal navigation [14,15]. The interaction with the substrate, i.e. the surface over which neurons are cultivated influences neuron differentiation, morphology, adhesion and axon or neurite outgrowth [1,14,16–21]. Neurons cultured on elastomeric materials such as polyacrylamide, agarose gels and polydimethylsiloxane (PDMS) showed increased branching [22,23] and extension [20,24] but the response to mechanical properties of the substrate varies considerably among different neuronal types [25]. PDMS is an elastomeric material widely used because of its biocompatibility, transparency and ease of fabrication [14,26–29].

GCs navigation such as growth, retraction, turning and branching are regulated by the dynamic reorganization of actin filaments and microtubules, which are linked by a complex biochemical machinery to guidance molecule receptors [1]. GCs integrate complex physical cues and grooves with a depth from 200 nm to some µm influence neurite growing direction [18,29–32]. The alignment or outgrowth increase is not observed on grooves less than 200 nm deep [1].

Paxillin is a focal adhesion-associated, tyrosine phosphorylated Src substrate, participating in several signaling pathways. Paxillin contains a number of motifs that mediate protein-protein interactions and serve as docking sites for cytoskeletal proteins, tyrosine kinases, serine/threonine kinases, GTPase activating proteins and other adaptor proteins that recruit additional enzymes into complexes with Paxillin [33–35]. The function of Paxillin includes also the regulation of cell spreading and cell motility [33,36].

In the present manuscript we analyze the combination of chemical cues and of nanopatterns on GC navigation. We cultivated dissociated hippocampal neurons from P2-P3 rats over PDMS substrates with patterned lines varying from 100 to 600 nm in height and we applied guidance cues, i.e. a stream of Netrin-1 molecules, so to combine a mechanical and a chemical cue. We also analyzed whether axons and dendrites respond differently to mechanical cues: axons were distinguished from dendrites because of the presence of specific neurofilaments in their cytoskeleton. In addition, we examined the expression and cell distribution of the focal adhesion-related protein Paxillin when neurites cross nanostructured lines and/or grow along them. Our results show that thin and highly motile structures such as filopodia climb over high obstacles in response to chemical cues in just a few minutes, but larger neuronal structures are less prompt and can climb similar obstacles but within hours or days.

## Methods

### Fabrication of soft PDMS substrates

Nanopatterned substrates were fabricated according to a previously established method [27]. Briefly, silicon moulds with lines of 2 µm width and 4 µm period were produced by UV photo-lithography and dry etching in a STPTS ICP reactor; depth of 100, 300 or 600 nm were obtained by tuning the application time of unswitched and continuous etching. Substrates for cell culture were obtained by mold casting of Sylgard 184 PDMS elastomer (Dow Corning, Midland, MI). The latter consists of a base and a curing agent, mixed at 1:10 (w) ratio and degassed for at least 30 minutes under vacuum. 1.5x1.5 cm^2^ silicon master with the selected topography were used as molds. The stack of glass/PDMS/mould was kept at 150 kPa of pressure and the temperature was raised to 120°C for 1h for curing. After cooling the system to RT, the PDMS substrates were peeled off from the masters. The height of the samples was measured with AFM and SEM (Figure S1).

### Neuronal culture preparation

Hippocampal neurons from Wistar rats (P2-P3) were prepared in accordance with the guidelines of the Italian Animal Welfare Act and their use had been previously approved by the Local Veterinary Service, by SISSA Ethics Committee board and by the National Ministry of Health (Permit Number: 630-III/14), as they are in accordance with the European Union guidelines for animal care (d.1.116/92; 86/609/C.E.). Animals were anesthetized with CO_2_, sacrificed by decapitation and all efforts were made to minimize suffering. Dissociated cells were plated at the concentration of 10^5^ cells/ml on polyornithine/matrigel precoated PDMS substrates in minimum essential medium (MEM) with Earle’s salts and GlutaMAX^TM^ supplemented with with 5% fetal calf serum (all from Invitrogen, Life Technologies, Gaithersburg, MD, USA), 0.5% D-glucose, 14 mM Hepes, 0.1 mg/ml apo-transferrin, 30 µg/ml insulin, 0.1 µg/ml D-biotin, 1 mM vitamin B12, and 2 µg/ml gentamycin (all from Sigma-Aldrich, St. Louis, MO). Neuronal cultures were maintained in an incubator at 37°C, 5% CO_2_ and 95% relative humidity and were used after 1 or 3 days of culture. Before starting the turning assay experiment, cultures were bathed in Ringer’s solution (145 mM NaCl, 3 mM KCl, 1.5 mM CaCl_2_, 1 mM MgCl_2_, 5 mM glucose, 10 mM HEPES, adjusted to pH 7.4 with NaOH; all from Sigma-Aldrich, St. Louis, MO).

### Immunostaining and imaging

Cells were fixed in 4% paraformaldehyde containing 0.15% picric acid in phosphate-buffered saline (PBS), saturated with 0.1 M glycine, permeabilized with 0.1% Triton X-100, saturated with 0.5% BSA (all from Sigma-Aldrich, St. Louis, MO) in PBS and then incubated for 1h with primary antibodies: mouse monoclonal anti-MAP2 antibody (Sigma-Aldrich, St. Louis, MO), mouse monoclonal anti-Paxillin antibody (Santa Cruz Biotechnology, Santa Cruz, CA), SMI 312 mouse monoclonal antibody (Covance, Berkeley, CA), and rabbit polyclonal anti-β-tubulin III antibody (Sigma-Aldrich, St. Louis, MO). The secondary antibodies were: goat anti-mouse immunoglobulin (Ig) G_1_-FITC and IgG_2a_-TRITC (Southern Biotech, Birmingham, AL), goat anti-rabbit 488 Alexa, goat anti-mouse 594 Alexa and biotinylated goat anti-rabbit. F-actin was marked with Alexa Fluor 488 phalloidin, biotin was recognized by Marina Blue-Streptavidin (all from Invitrogen, Life Technologies, Gaithersburg, MD, USA) and the incubation time was 30 min. All the incubations were performed at room temperature (20-22°C). The cells were examined using a Leica DM6000 fluorescent microscope equipped with DIC and fluorescence optics, CCD camera and Volocity 5.4 3D imaging software (PerkinElmer, Coventry, UK). The fluorescence images were collected with a 63x magnification and 1.4 NA oil-immersion objective. For quantification analysis of the Paxillin staining, neurons were plated on 600 nm high lines and fixed after 3 days of culture. For each image at least 20 slices were acquired at a slice spacing of 0.5 µm and also DIC images were acquired to visualize the pattern. Region of interest (ROI) was traced along the edges of neurites using DIC and tubulin staining as reference. The ratio of mean fluorescence intensity of Paxillin staining between aligned and crossing neurites was calculated. In addition, the ratio between surface area relative to Paxillin staining and total surface area of selected neurite was extracted for each selected neurite. Volocity 5.4 3D imaging software and Image J by W. Rasband (developed at the U.S. National Institutes of Health and available at http://rsbweb.nih.gov/ij/) were used for image processing.

### GC turning assays

Dissociated hippocampal neurons were examined 24 hr after plating. A stable Netrin-1 (R&D Systems, Minneapolis, MN) gradient was created and maintained using a Picospritzer III (Intracel, Royston, UK) ejecting picoliter pulses of Netrin-1 solution (300 ng/ml in PBS supplemented with 0.1% BSA) applied repetitively at a pressure of 7 psi, frequency of 2 Hz and duration 20 ms from a micropipette with 1 µm opening. The micropipette tip was positioned 100 µm from the GC center. Cells were maintained at 37°C and atmospheric CO_2_ on a heated stage in a Ringer’s solution and examined up to 1 hr after the onset of the Netrin-1 gradient for each coverslip. Cell images were recorded every 60 s. GCs were examined up to 1 hr in a Ringer’s solution after the onset of the Netrin-1 gradient in all experiments. In control experiments, PBS alone was ejected from the micropipette. The neurite trajectories at 30 min after the onset of the Netrin-1 gradient were traced from the video images.

### Statistical analysis

Data are shown as means ± sem and were collected from at least three independent experiments. Statistical significance was evaluated using the chi-square test, Student’s t-test and ANOVA using the Bonferroni correction. A *p* value with α=0.05 was extracted for each sample combination.

## Results

Hippocampal neurons were dissociated from P2-P3 rats and were plated on flat PDMS substrates or on PDMS substrates with patterned lines 2 µm wide with a period of 4 µm and 100, 300 or 600 nm high. Therefore neurites emerging from the soma of dissociated neurons could either grow following the lines or climb a step with a height in the hundred-nanometer range. In the present manuscript we investigated the effect of the step height in the turning assays [37,38] and on neurite growth.

### Turning assays in the presence of steps of varying heights

Neurites protruding from hippocampal neurons plated on PDMS substrates with patterned lines 100 nm high grew randomly in all directions and crossed these steps spontaneously 1-3 days after plating.

**Figure 1:**
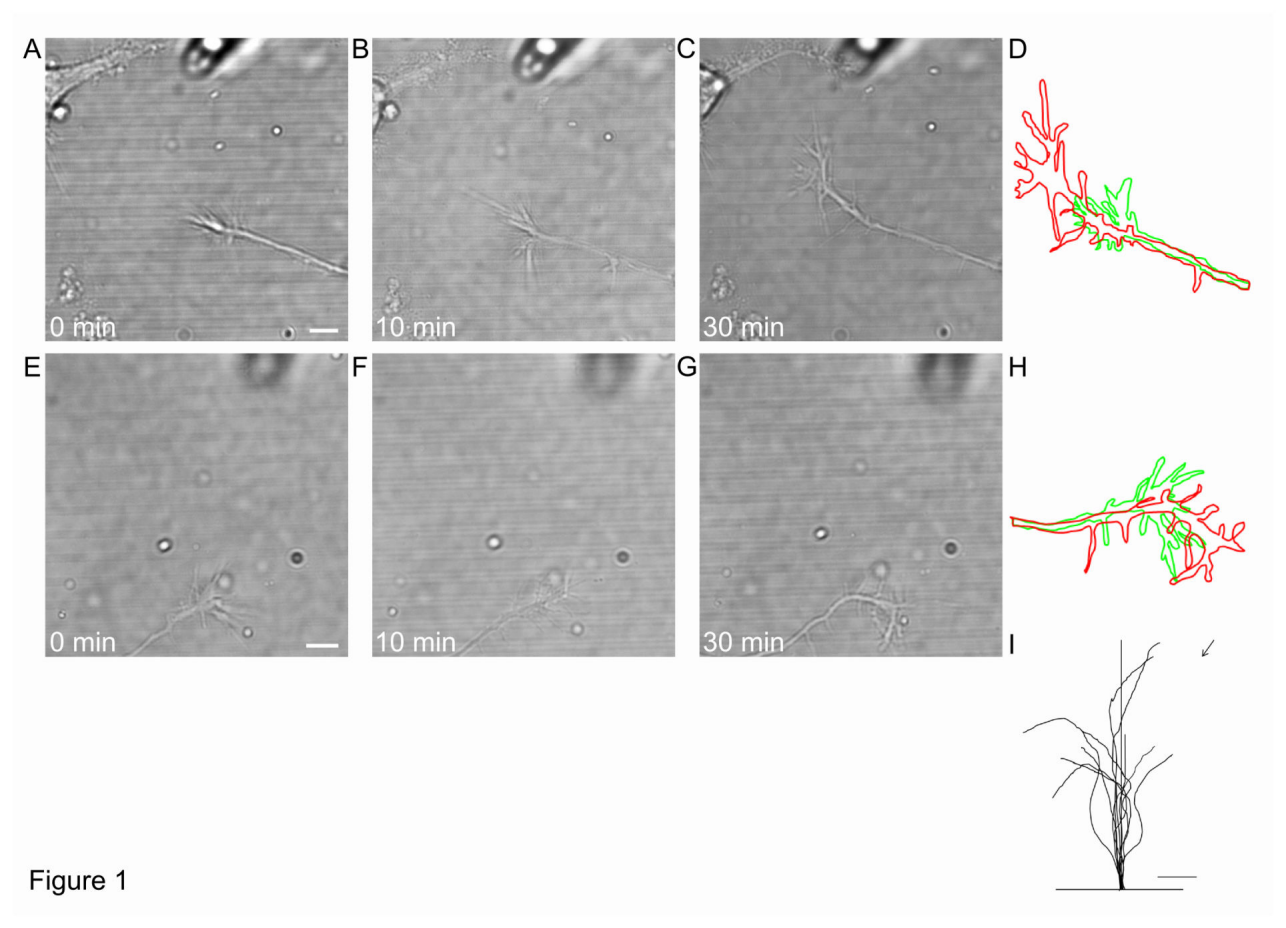
Hippocampal GC’s on 100nm high PDMS lines are both attracted and repelled by Netrin-1. (**A**–**C**) DIC images of the GC 24 after plating on PDMS lines 100 nm high at onset/0 min (A), 10 min (B) and 30 min (C) after exposure to 300 ng/ml Netrin-1 gradient. (**D**) GC profiles at 0 min (green) and 30 min (red) for the GC shown in (A–C). (**E**–**G**) same as (A–C) but for the GC repelled by Netrin-1. (**H**) GC profiles at 0 min (green) and 30 min (red) after exposure to Netrin-1 gradient for the GC shown in (E–G). (**I**) Traces of 10 individual GC trajectories 30 min after Netrin-1 exposure. The 20 µm segment of the neurite is also shown. Scale bar, 5 µm.

We performed growth cone (GC) turning assays 24 h after plating. In these experiments, a 300 ng/ml concentration of Netrin-1 was ejected through a conventional glass patch pipette with a hole of 1 µm in diameter and the GC response immediately after the initiation of the Netrin-1 flow was monitored with videoimaging (Figure 1). In more than one third of these experiments (12/28) the GC changed its growth direction and grew toward the pipette (Figure 1B and C) within 10-30 minutes. In other experiments (13/28) GC completely changed its growing direction and turned away from the source of the Netrin-1 gradient, showing repulsion (Figure 1 D–F). During the 1 h observation, GCs were able to cross up to 6 lines and the maximum neurite extension was 18,4 µm (considering the change of position of the GC center from 0 min to 1 h of Netrin-1 exposure). In the presence of Netrin-1 gradient, GC’s filopodia moved rapidly across the 100 nm lines in all directions, i.e. towards and away from the pipette (Figure 1 B, F and Video S1).

In 3 out of 28 experiments neither attraction nor repulsion was observed and no GC crossed the lines during the duration of the experiment, i.e. up to one hour. Therefore the GC behavior in the presence of guidance molecules and neurite growth over patterned lines 100 nm high is similar to what observed when neurons are plated over flat surfaces (p=0.64 and [39]). These experiments confirmed that Netrin-1 can both attract and repel hippocampal GCs [38,40] as summarized in Figure 1I. When a vehicle replaced Netrin-1 inside the suction pipette, the GC navigation was not affected (Figure S2, p=0.90).

**Figure 2:**
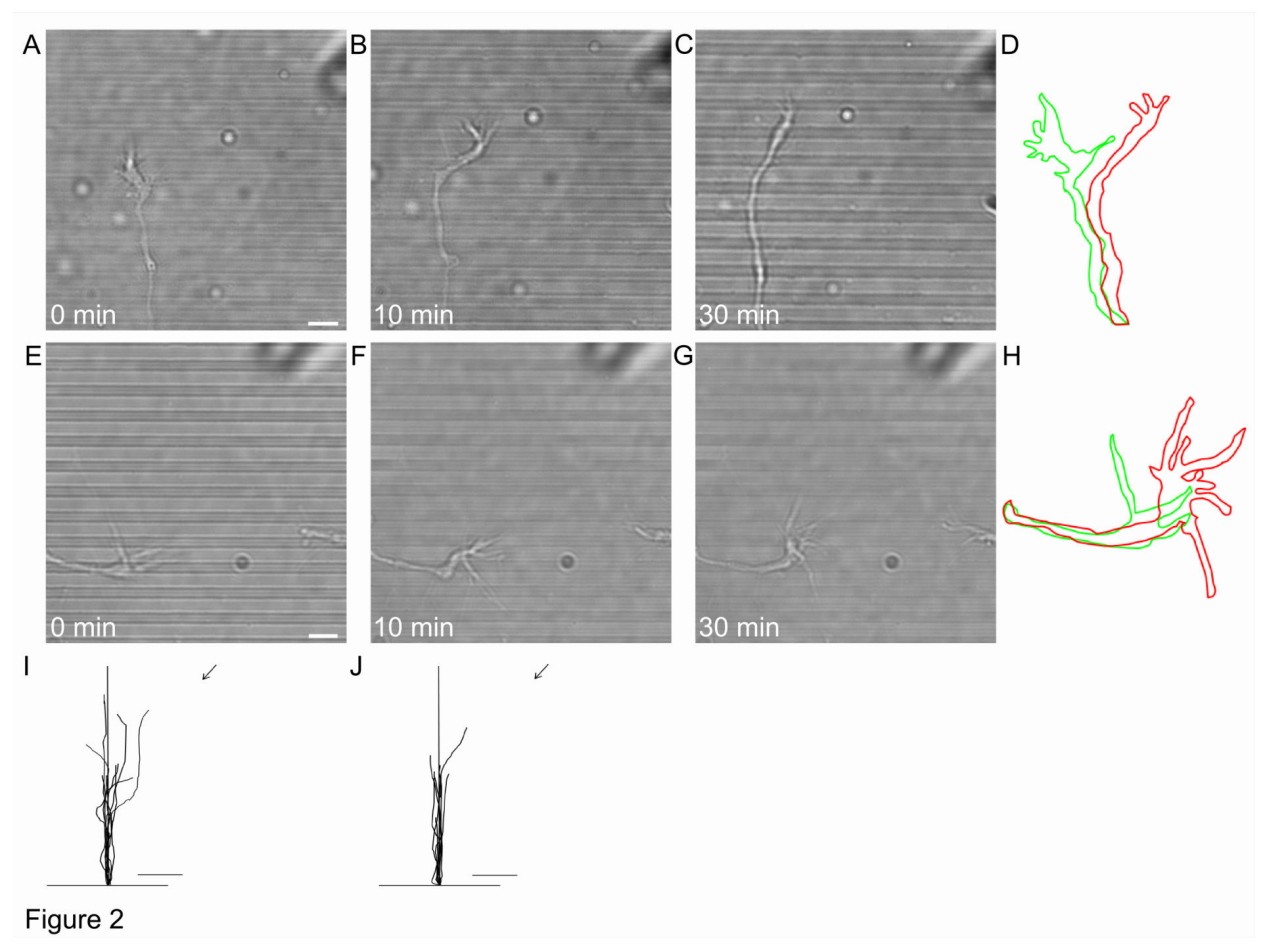
In the presence of Netrin-1, GCs occasionally cross PDMS lines 300 nm and 600 nm high. (**A**–**C**) DIC images of the GC 24 h after plating on PDMS lines 300 nm high at onset (A) and after 10 min (B) and 30 min (C) exposure to 300 ng/ml Netrin-1 gradient. (**D**) GC profiles at 0 min (green) and 30 min (red) for the GC shown in (A–C). (**E**–**G**) DIC images of the GC 24 h after plating on PDMS lines 600 nm high at onset (E) and after 10 min (F) and 30 min (G) exposure to Netrin-1 gradient. (**H**) GC profiles at 0 min (green) and 30 min (red) after exposure to Netrin-1 gradient for the GC shown in (E–G). (**I**, **J**) Traces of 10 individual GC trajectories 30 min after Netrin-1 exposure for lines 300 nm (I) and 600 nm (J) high. The 20 µm segment of the neurite is also shown. Scale bar, 5 µm.

We then repeated the same experiments with patterned lines and with higher steps. In the presence of steps of 300 nm, neurites grew along the lines but could only occasionally climb over these steps (Figure 2A). Filopodia emerging from GCs at the neurite tips explored the environment also climbing above 300 nm steps. Under these conditions, occasionally GC attraction (4/25) (Figure 2B) or repulsion (1/25) was observed but neurites could not follow the gradient of guidance molecules (22/25) within the duration of the turning assay (up to 1 hr). GCs were able to cross up to 4 steps with the maximum crossing distance of 12,4 µm. In the presence of steps 600 nm high, filopodia crossed these steps, while for GCs only attraction (2/19) but no repulsion was observed. GCs could cross up to 2 steps with the maximum crossing distance of 8,5 µm. We never observed neurites climbing over these steps during exposures to a Netrin-1 up to 1h (n=19). Under these conditions, neurites continued to grow along the lines (Figure 2D–F and J).

**Figure 3:**
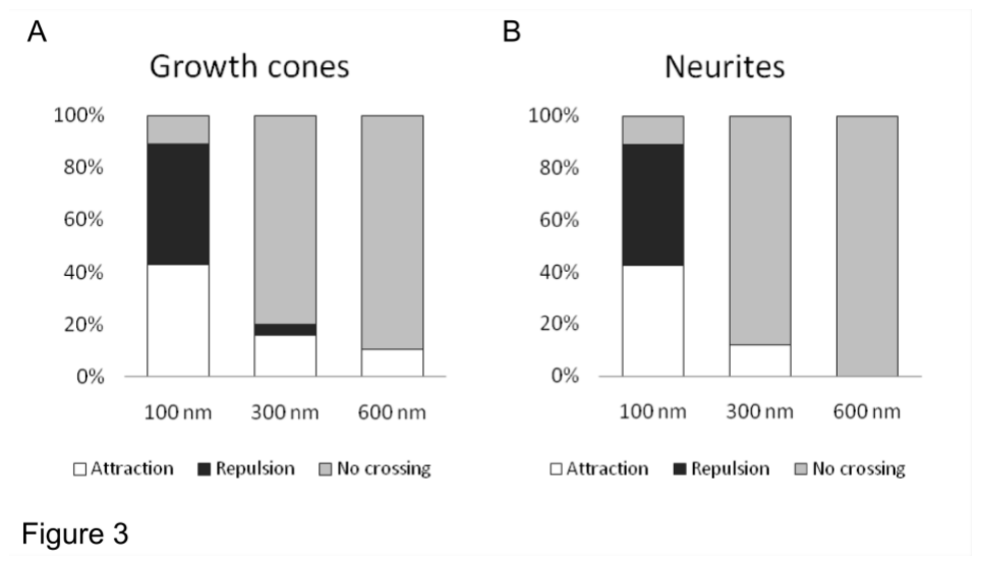
Summary of the GC turning experiments performed on PDMS substrates patterned with 100 nm (n=28), 300 nm (n=25), and 600 nm high lines (n=19) and up to 1h exposure to 300 ng/ml Netrin-1 gradient. (**A**) GC and (**B**) neurites response was classified as attraction (white boxes), repulsion (black boxes) or no crossing (grey boxes). The duration of turning experiments was up to 1 hr.

Collected data from 72 experiments (Figure 3) show that neurites grown over patterns of lines with a height of 100 nm could cross these steps in response to guidance molecules and could exhibit both attraction and repulsion behaving as on flat surfaces (25/28, p=0.32 and [39]). In contrast, when the step height was 300 or 600 nm, only GCs and their filopodia could climb over these steps and follow the gradient of guidance molecules but with significantly lower frequency (5/25 for 300 nm and 2/19, for 600 nm, p<0.01, Figure 3A). The reduced ability to cross the higher lines was even more pronounced for neurites: only very occasionally (5/25, p<0.01) neurites were seen crossing a step of 300 nm, but never a step of 600 nm (0/19, p<0.01) during 1 hr of exposure to Netrin-1 (Figure 3B).

### Dendrites and axons

Our turning experiments lasted up to 1 hr and, in this period of time, filopodia and - to a lesser extent - GCs, could climb obstacles in response to chemical cues. Therefore, we asked whether neurites could climb high lines in a longer period of time and therefore we cultured the same hippocampal neurons for 3 days on the same patterns. Axons and dendrites are not specified when they arise [41] but when neurons are cultured for more than 3 days it is possible to selectively label axons and dendrites. Therefore we used the axonal neurofilament marker SMI 312 [42–44] and the microtubule-associated protein (MAP) 2 [45,46]. After 1 or 2 days of culture we could not classify reliably axons and dendrites using these markers since most of the neurons were not fully polarized. After 3 days of culture this identification became more evident: SMI 312 – positive axons were detected in 56.7 ± 2.7% of cells (858 cells analyzed) and 90.4 ± 2.6% (479 cells analyzed) of neurons expressed MAP2. However, with MAP2, it was not possible to selectively stain dendrites as all neurites were found to be MAP2-positive, including nascent axons (Figure S3). After 3 days of culture, not all hippocampal P2-P3 neurons were fully polarized and dendrite identification and maturation require longer periods of culture. Indeed, hippocampal E18 neurons show significant dendritic growth only after 4 days of culture, i.e. 2-3 days after axonal outgrowth [41,46].

**Figure 4:**
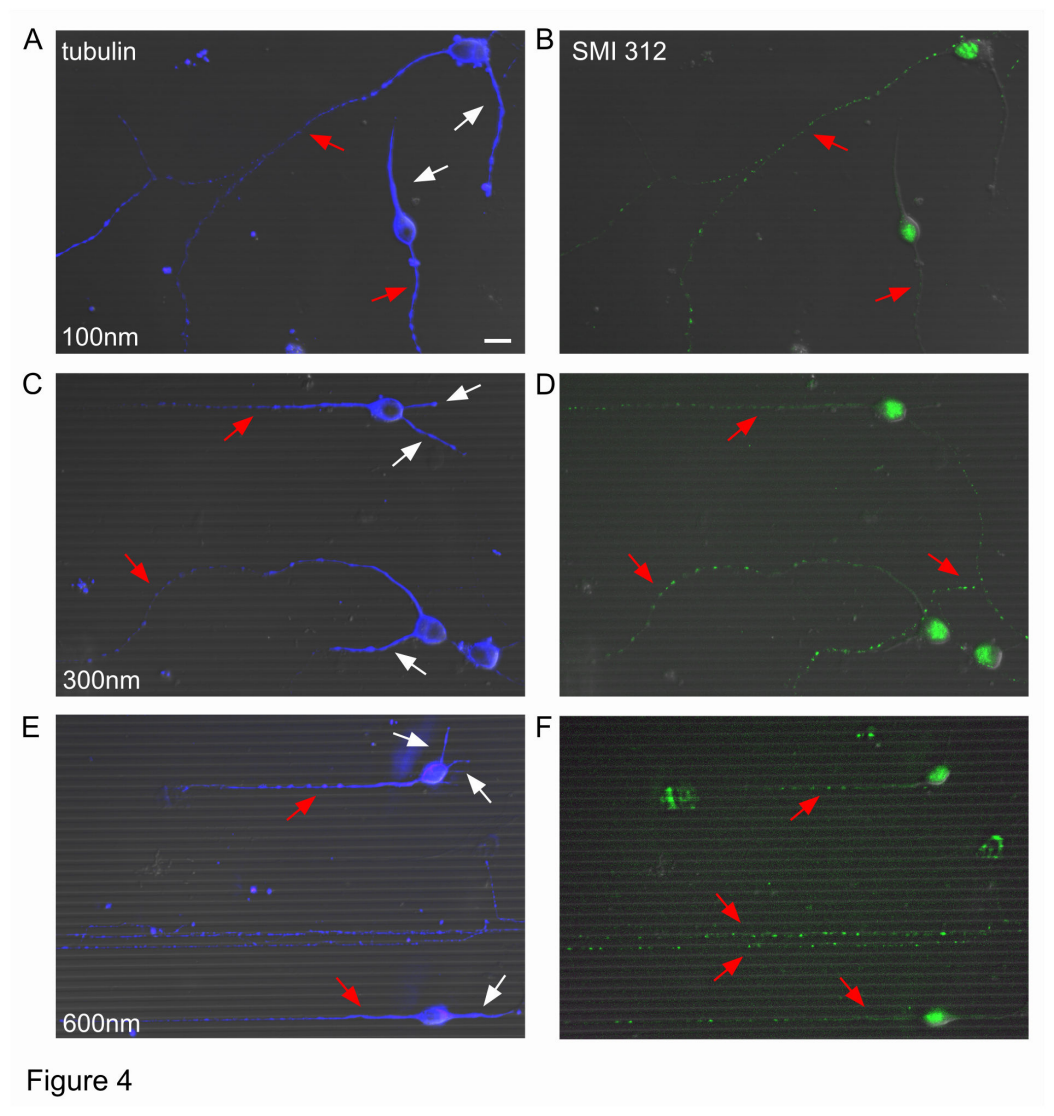
Expression of tubulin and axonal neurofilaments after 3 days of culture. Overlapped DIC and fluorescence images of neurons stained with anti-β-tubulin III antibody (blue) and axonal neurofilament marker SMI 312 (green) for 100 nm (**A**–**B**), 300 nm (**C**–**D**) and 600 nm (**E**–**F**) high lines. Red arrows indicate axons, white ones dendrites. Scale bar, 10 µm.

When neurons were grown on patterned substrates for 3 days, we observed a significant proportion (around 57%) of neurons positive for SMI 312, a marker of axons. When neurons were cultivated over 100 nm high lines, both axons and β-tubulin III-positive neurites were able to cross these lines easily (Figure 4 A-B). Axons and dendrites could cross also 300 nm (Figure 4 C-D) and 600 nm lines (Figure 4 E-F), but, progressively, to a lower extent. We observed also neurites growing along a line for some µm and subsequently crossing the line and *vice versa*, i.e. neurites initiating their growth crossing a line and then aligning to it.

**Figure 5:**
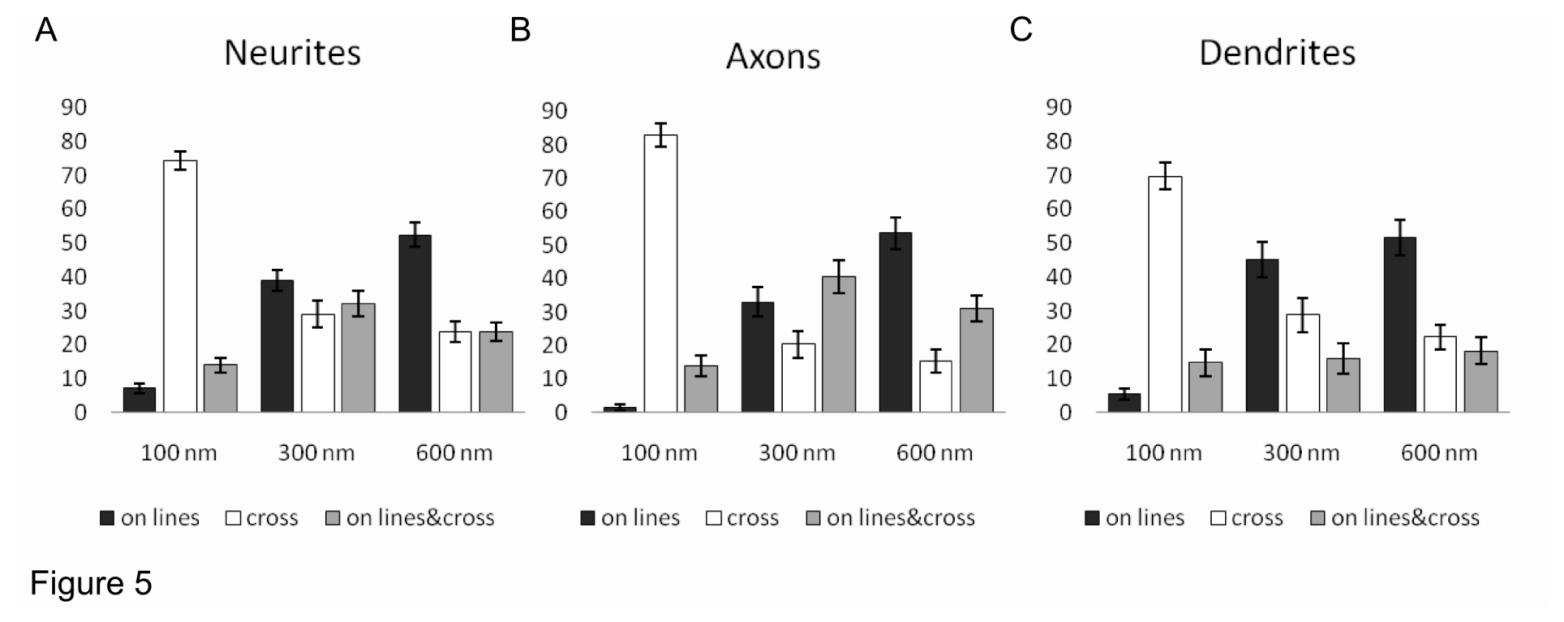
Summary of neurite growth on PDMS patterned substrates after 3 days of culture. Data were obtained from immunofluorescence images of neurons stained for β-tubulin III and SMI 312 after 3 days of culture. (**A**) Neurites counted from all β-tubulin III-positive neurons, (**B**) SMI 312-positive axons (expressed in 57% of total neurons) and (**C**) Dendrites (i.e. neurites of SMI 312-positive neurons excluding the axons) counted on 100 nm, 300 nm and 600 nm high lines. Neurites grew on lines (black boxes), crossed the lines (white boxes) or grew both parallel to lines and crossing the lines (grey boxes). The statistical significance was evaluated using ANOVA (see Methods).

Collected data are summarized in Figure 5: on 100 nm lines, the majority of neurites crossed lines (80.1 ± 2.6%, p<0.05) and this behavior was more evident for axons compared to the dendrites (84.3 ± 3.3% and 77.8 ± 4.0% respectively, p<0.05). The proportion of aligned neurites on 100 nm lines was significantly lower (5.5 ± 1.4%, p<0.05) and axons did not differ from dendrites (1.6 ± 0.9% and 5.9 ± 1.7% respectively, p=0.1). In the presence of higher ridges, the percentage of aligned neurites significantly increased (38.9 ± 3.0% for 300 nm lines and 52.5 ± 3.6%, for 600 nm lines respectively, p<0.05). Axons and dendrites aligned equally well on both 300 nm lines (42.2 ± 4.2% versus 50.2 ± 5.2%, p=0.09) and on 600 nm lines (53.5 ± 4.6% versus 56.1 ± 5.1, p=1). Consequently, the percentage of neurites crossing lines significantly reduced together with their increasing height (29.0 ± 3.9% for 300 nm lines and 23.8 ± 3.0% for 600 nm lines respectively, p<0.05). Axons and dendrites crossed those lines with comparable frequency (21.1 ± 4.1% versus 32.0 ± 5.1 for 300 nm, p=0.47 and 15.5 ± 3.5% versus 24.1 ± 3.7 for 600 nm lines, p=0.49 respectively).

### Climbing steps and adhesion

When neurites climb over obstacles, such as the lines used here, adhesion between the growing neurite and the substrate must take place. Adhesion requires the coordinated action of the extracellular matrix, the integrins and the cell cytoskeleton that interact at specific sites called focal contacts [47]. Focal contacts are dynamic groups of structural and regulatory proteins transducing external signals, such as mechanical and biochemical cues [48,49]. Several tens of distinct proteins form these focal contacts [35] and Paxillin - which is a focal adhesion-associated protein via integrin - has to be phosphorylated to become active [33].

**Figure 6:**
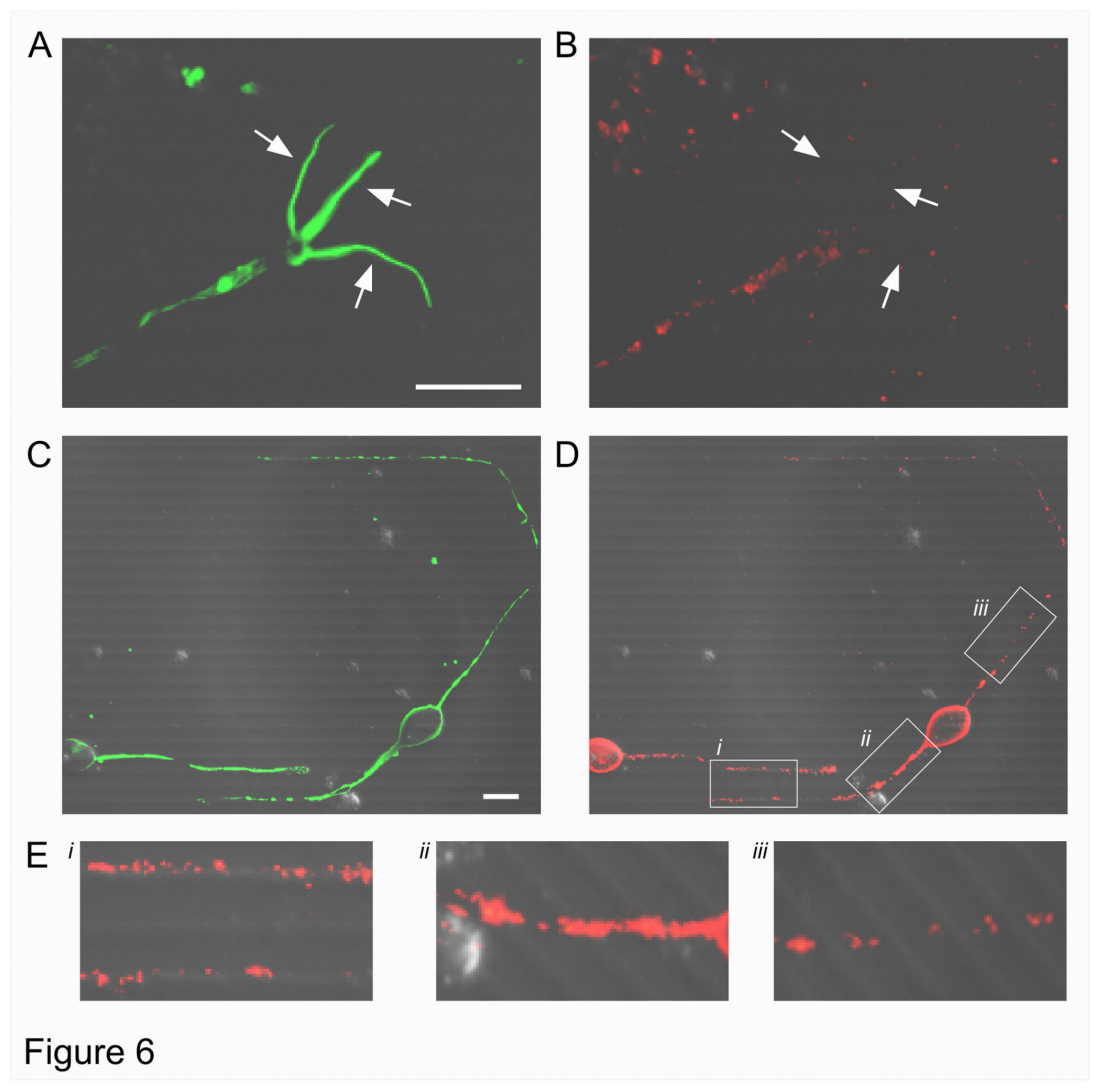
Expression of Paxillin on PDMS patterned substrates. After 1 day of culture, GCs were labeled for F-actin (**A**) and Paxillin (**B**). Arrows indicate filopodia that are Paxillin-negative. After 3 days, neurites were labeled for β-tubulin III (**C**) and Paxillin (**D**) respectively. (**E**) Insets (*i*, *ii* and *iii*) shown in D for aligned and crossing neurites. Neurites aligned to lines, grow mostly at the top of the steps and have punctate Paxillin staining. When neurites cross lines Paxillin staining is also seen at the bottom of these steps. DIC images are merged with fluorescence images. Scale bar, 10 µm.

We considered the expression of Paxillin on PDMS lines 24 h after plating, the same time-window that corresponds to GC turning experiments where we observed high motility of filopodia, capable to cross steps regardless of their height, while GCs and neurites were progressively slower when their height increased. As shown in Figure 6, hippocampal neurites [25] and GCs in their central domain express Paxillin whereas filopodia, stained for F-actin (Figure 6A), are Paxillin-negative (Figure 6B). Similarly to migrating cells, where during migration cell-substrate adhesions are absent or only transiently identifiable and adhesion sites must be disrupted for a cell to move [50], our observations explain in part why filopodia can climb easily over obstacles.

Finally we investigated the expression of Paxillin and β-tubulin III after 3 days of culture, when neurons become more mature. Neurites preferentially grow along the lines and more often on top of them (see Figure 4E-F and Figure 6). Neurites, both axons and dendrites, growing on top of a line have punctuated staining of Paxillin (Figure 6D), stronger on thicker neurites near the soma and progressively less strong on thinner and distal neurites. In the presence of lines with a height of 600 nm, neurites rarely cross, but when they cross staining for Paxillin is found both at the bottom and at the top of the lines (compare insets of Figure 6D-E). We quantified the Paxillin staining of the aligned and crossing neurites. The ratio of the mean fluorescence intensity between aligned and crossing neurites was 0.99 ± 0.03 (n=36) indicating no significant difference (p=0.43). Moreover, the ratio of Paxillin–positive surface area to total surface area of the corresponding neurite was 0.49 ± 0.04 for aligned neurites (n=24) and 0.45 ± 0.05 (n=14) for crossing neurites (p=0.40).

## Discussion

In the present manuscript we have analyzed how developing neurites respond to the combination of chemical and mechanical cues. We have performed turning experiments with Netrin-1 gradients on hippocampal GCs 24 h after plating on PDMS substrates patterned with lines 100 nm, 300 nm and 600 nm high. Our turning experiments had up to 1 hr duration, providing information on a short and medium range time scale. We also cultured the neurons on the same nanopatterned substrates for 3 days, so to analyze the effect of mechanical cues on a longer time scale. Our results indicate that: i - in response to chemical cues, filopodia can climb over obstacles in minutes; ii- GCs and neurites respond to chemical cues less promptly; iii - on a time scale of hours and days also neurites can climb over high lines; iv- neurites preferentially grow parallel to lines higher than 300 nm and preferentially on top of them; v - when neurites cross over obstacles higher than 300 nm, focal adhesion occurs both at the bottom of encountered obstacles and at the top. These results show that neurites respond in a complex way to the combination of biochemical and mechanical cues depending on the time scale and properties of applied cues.

### The combination of chemical and mechanical cues

As shown in Figures 1-2, when a Netrin-1 gradient is applied to neurons grown over patterned PDMS substrates with lines 600 nm high, filopodia but not neurites can be attracted or repelled by the chemical gradient. When the height of the lines was reduced to 300 nm it was possible to observe occasionally both GCs attraction and repulsion but more often (20/25) GCs did not modify their navigation in response to the applied chemical gradient. In the presence of lines 100 nm high both attraction and repulsion was observed and almost 90% (25/28) of GCs showed response to the applied Netrin-1 gradient. When neurons are grown for 3 days on the same substrates (with lines of the same height), neurites are able to cross also line 300 and 600 nm high, as shown in Figures 4 and 5.

The observation that neurites grown over patterned lines 600 nm high do not respond to biochemical gradients as neurites grown over lines lower lines do, could be explained by a screening effect of the lines, so that the guidance molecules delivered through the pipette do not reach the GC. Three observations argue against this possibility: firstly, during the turning assays filopodia are seen crossing over lines 600 nm high (see Figure 2) and hippocampal filopodia are decorated with receptors for guidance molecules such as Netrin-1 (Figure S4 and [39]); secondly, in the presence of a similar chemical gradient of actin depolymerizing agents, such as Latrunculin A, GCs of neurons grown on lines 300nm or 600 nm high stop their exploratory motion very quickly and their filopodia collapse, as it happens in a flat dish (Video S2 and [51]) and thirdly, diffusional paths of Netrin-1 molecules would have a micrometric-scale dimensions with estimated total volume explored by the molecule per unit time around 5 µm^3^s^-1^ [52] therefore it is theoretically improbable to have screening effect even with the highest lines, i.e. 600 nm.

Our results indicate that the increase of the line height significantly reduces the ability of neurites to cross them: on 100 nm lines, neurites can grow for several tens of µm during the Netrin-1 gradient exposure, while on 600 nm lines they are not able to grow and cross lines in the same time interval (compare Figures 1A–C and 2D–F). After 3 days of culture 29% and 24% of neurites can cross both the 300 and 600 nm lines, suggesting that crossing neurites require a longer time to grow and climb over higher lines while 39% and 53% of neurites grow along the 300 nm and 600 nm lines respectively.

Filopodia are highly motile structures continuously exploring the environment and extending or lifting up by several µm in a minute [39,53] and it is not surprising that they can respond quickly to a chemical gradient also in the presence of obstacles. GCs formed by lamellipodia and filopodia are more bulky and can have a significant motility but less pronounced than filopodia [39] while neurites navigation occurs on a longer time scale, i.e. hours and days [41].

### Axons versus dendrites

During polarization, axons and dendrites develop morphologically and functionally specific compartments that differ from each other in the composition of their proteins and organelles. Axon specification is regulated by signaling molecules that have established roles in cytoskeletal rearrangements and protein trafficking [54]. Microtubule-associated proteins (MAPs) of the MAP2/Tau family stabilize and regulate microtubule networks [46]. Shortly after axonogenesis in developing hippocampal neuronal cultures, Tau gradually segregates into axons, while MAP2 segregates into the nascent dendrites (at this stage dendrite precursors are called ‘minor neurites’). At this stage, axon can be recognized as the longest neurite, but sometimes this neurite can retract and become dendrite [41]. For this reason, we used immunofluorescence assays for axons and dendrites markers. Both Tau and MAP2 were tested: however, Tau expression was not restricted to axons (unpublished observations) and MAP2 was not specific for dendrites at this stage (see Figure S3). This is in agreement with what was previously observed with embryonic chick neurons [55]. We used the axonal neurofilament marker SMI 312 that was selectively expressed in the axons of 57% of the neurons after the 3 days of culture and we considered as dendrites only the SMI 312-negative neurites of these SMI 312 expressing neurons. Axons and dendrites were able to cross or grow along lines equally well (Figures 4 and 5). Taken together, these observations suggest that, at this developmental stage, axons and dendrites do not seem to differ in their ability to cross the obstacles.

### Line crossing

Both groove floors and plateaus present distinct topographical cues to cells [1]. Our substrates have micrometric width and period (2 µm and 4 µm respectively) and differ in height at the nanometer scale, i.e. subcellular scales. Cell bodies are too large to sit inside the grooves, whereas neurites can grow inside the lines although they prefer to grow on the top of the lines (Figure 4E and [56,57]) instead of growing in the grooves [58] or perpendicularly [28,31].

We observed no Paxillin expression in filopodia (Figure 6B) and the absence of staining of this adhesion marker is consistent with the high motility of filopodia. Actin dynamics is particularly important for exploration of the environment [3,15] and filopodia ability to climb over obstacles, at least initially, seems to be adhesion-independent. After 3 days of culture, neurites grew more frequently parallel to the lines 300 and 600 nm high and, in particular, adhesion at the roofs of these lines was preferable (Figure 6). Neurites that crossed those lines showed no difference in Paxillin expression compared to aligned neurites. These observations suggest that on longer time-scales also adhesion-dependent machinery is involved in climbing, together with the cytoskeleton components - such as spectrins, actin filaments, microtubules, neurofilaments - , motor proteins and signaling pathways [15,47]. Interestingly, recent evidence suggests that guidance molecules may direct axon pathfinding by controlling integrins-based adhesion [59] since Netrin itself can directly bind integrin receptors [60,61]. The complex coordination between biochemical signals and receptor adhesion is emerging as an important regulatory mechanism to control and determine neuronal navigation, i.e. whether the neurite will climb over the obstacle.

## Supporting Information

Figure S1 **Measure of the steps height with AFM and SEM.** (A) from left to right: AFM height image of 100, 300 and 600 nm high PDMS lines respectively. (B) Height profiles obtained from the AFM images of the 100, 300 and 600 nm high lines respectively. (C) SEM images of the silicon masters used as template for the fabrication of PDMS substrates with 100, 300 and 600 nm high lines respectively. AFM and SEM were used as described in [27]. Scale bar, 5 µm.
(DOCX)

Figure S2 **Control experiments with PBS.** (A) from left to right: DIC images of the GC 24 after plating on PDMS lines 100nm high at 0 min, 10 min and 20 min after exposure to PBS. (B) same as A but for the 300 nm high lines. (C) same as (A) but for the 600 nm high lines. (D) Summary of the GC turning experiments performed on PDMS substrates patterned with 100nm, 300nm and 600nm high lines and up to 1h exposure to PBS. For each substrate, at least 15 cells were analyzed. GC and neurites response was classified as attraction (white boxes), repulsion (black boxes) or no response (grey boxes). Scale bar, 10 µm.
(DOCX)

Figure S3 **MAP2 is not selectively expressed in dendrites of hippocampal neurons after 3 days of cultures.** (A) DIC and (B) fluorescence image of MAP2-expressing neuron. MAP2 is expressed in all neurites emerging from the soma, including the longest (indicated by the arrow) likely to be an axon. Scale bar, 10 µm.
(DOCX)

Figure S4 **Hippocampal GCs and filopodia express Netrin-1 receptors DCC and UNC5A.** Fluorescence confocal images of a hippocampal GC 24 h after plating stained for DCC (A) and UNC5A (B). (C) Merged images. White dotted line indicates the GC outline. Scale bar, 5 µm.
(DOCX)

Video S1 **Video of hippocampal GC 24h after plating on PDMS lines 100 nm high and exposed to 300 ng/ml Netrin-1 gradient for 1h.** Images were acquired every 60 s. GC was observed with a 60x magnification and a 1.42 NA oil-immersion objective.
(MOV)

Video S2 **Video of hippocampal GC 24h after plating on PDMS lines 300 nm high 5 min before and 5 min after exposure to 100 nM Latrunculin A gradient.** Images were acquired every 30 s. GC was observed with a 60x magnification and a 1.42 NA oil-immersion objective.
(MOV)
