# Supplementary material for: Can Hippocampal Neurites and Growth Cones Climb over Obstacles?
Source: PLoS One. 2013 Sep 6;8(9):e73966. doi: 10.1371/journal.pone.0073966 (PMC3765352; doi:10.1371/journal.pone.0073966)
Supplement: Figure S1 — Measure of the steps height with AFM and SEM. (A) from left to right: AFM height image of 100, 300 and 600 nm high PDMS lines respectively. (B) Height profiles obtained from the AFM images of the 100, 300 and 600 nm high lines respectively. (C) SEM images of the silicon masters used as template for the fabrication of PDMS substrates with 100, 300 and 600 nm high lines respectively. AFM and SEM were used as described in [27]. Scale bar, 5 µm. (DOCX) [file pone.0073966.s001.docx]

**Supporting Information**


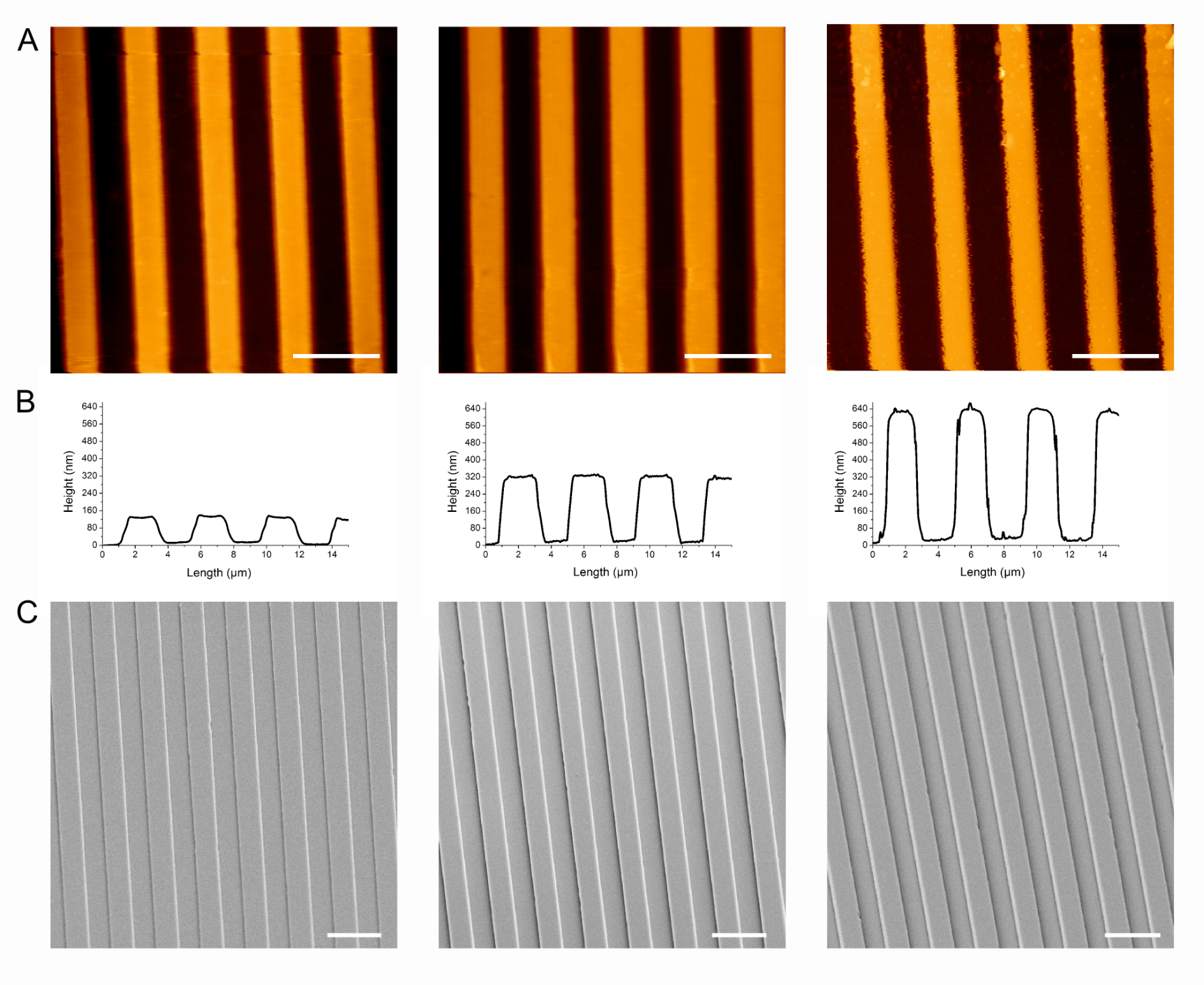


**Figure S1.** Measure of the steps height with AFM and SEM. (A) from left to right: AFM height image of 100, 300 and 600 nm PDMS lines respectively. (B) Height profiles obtained from the AFM images of the 100, 300 and 600 nm lines respectively. (C) SEM images of the silicon masters used as template for the fabrication of PDMS substrates with 100, 300 and 600 nm high lines respectively. AFM and SEM were used as described in [27]. Scale bar, 5μm.
