# Supplementary material for: Can Hippocampal Neurites and Growth Cones Climb over Obstacles?
Source: PLoS One. 2013 Sep 6;8(9):e73966. doi: 10.1371/journal.pone.0073966 (PMC3765352; doi:10.1371/journal.pone.0073966)
Supplement: Figure S2 — Control experiments with PBS. (A) from left to right: DIC images of the GC 24 after plating on PDMS lines 100nm high at 0 min, 10 min and 20 min after exposure to PBS. (B) same as A but for the 300 nm high lines. (C) same as (A) but for the 600 nm high lines. (D) Summary of the GC turning experiments performed on PDMS substrates patterned with 100nm, 300nm and 600nm high lines and up to 1h exposure to PBS. For each substrate, at least 15 cells were analyzed. GC and neurites response was classified as attraction (white boxes), repulsion (black boxes) or no response (grey boxes). Scale bar, 10 µm. (DOCX) [file pone.0073966.s002.docx]

**Supporting Information**


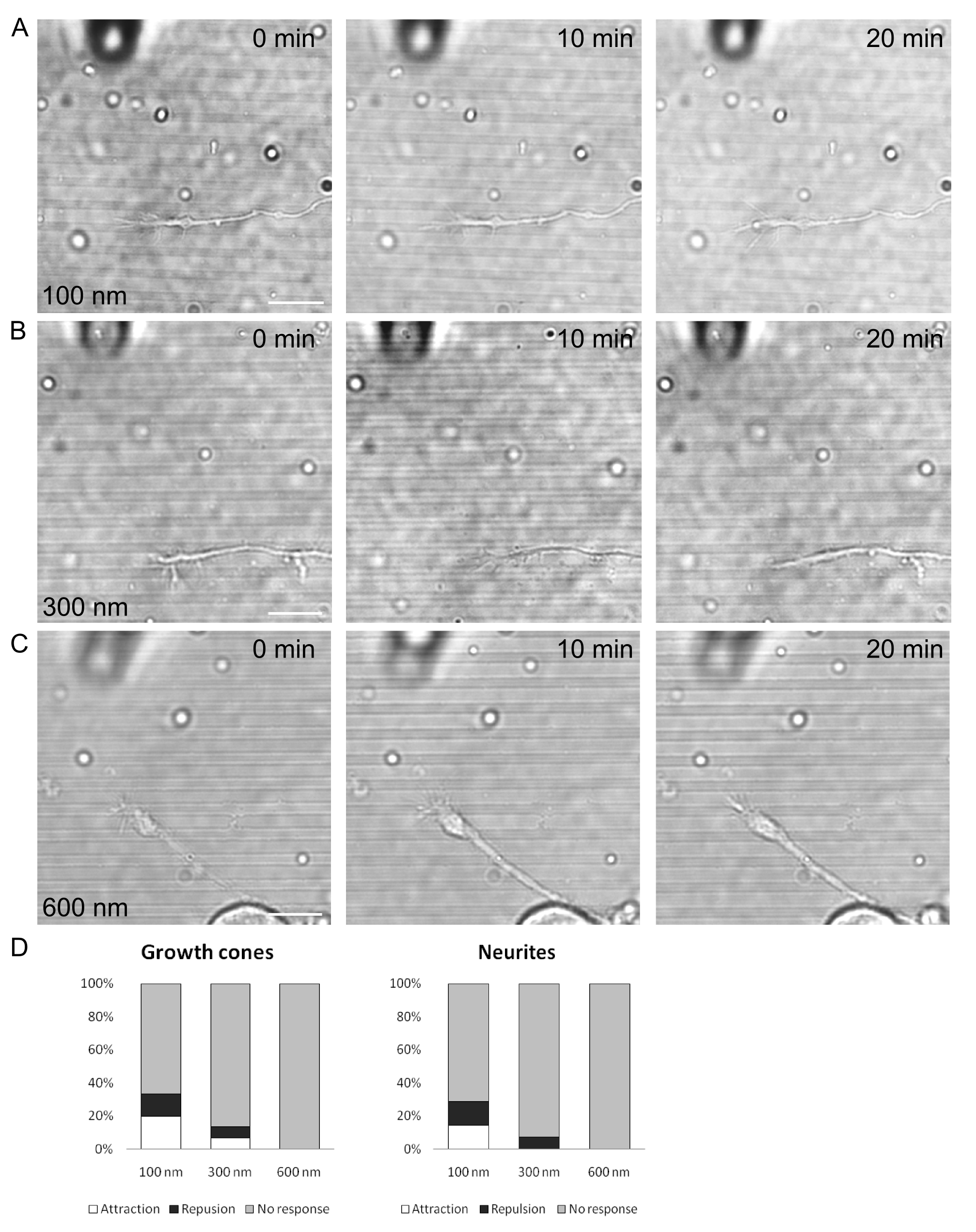


**Figure S2.** Control experiments with PBS. (A) from left to right: DIC images of the GC 24 after plating on 100nm PDMS lines at 0 min, 10 min and 20 min after exposure to PBS. (B) same as A but for the 300 nm lines. (C) same as (A) but for the 600 nm lines. (D) Summary of the GC turning experiments performed on PDMS substrates patterned with 100nm, 300nm and 600nm lines and up to 1h exposure to PBS. For each substrate, at least 15 cells were analyzed. GC and neurites response was classified as attraction (white boxes), repulsion (black boxes) or no response (grey boxes). Scale bar, 10 μm.
