# Supplementary material for: Can Hippocampal Neurites and Growth Cones Climb over Obstacles?
Source: PLoS One. 2013 Sep 6;8(9):e73966. doi: 10.1371/journal.pone.0073966 (PMC3765352; doi:10.1371/journal.pone.0073966)
Supplement: Figure S3 — MAP2 is not selectively expressed in dendrites of hippocampal neurons after 3 days of cultures. (A) DIC and (B) fluorescence image of MAP2-expressing neuron. MAP2 is expressed in all neurites emerging from the soma, including the longest (indicated by the arrow) likely to be an axon. Scale bar, 10 µm. (DOCX) [file pone.0073966.s003.docx]

**Supporting Information**


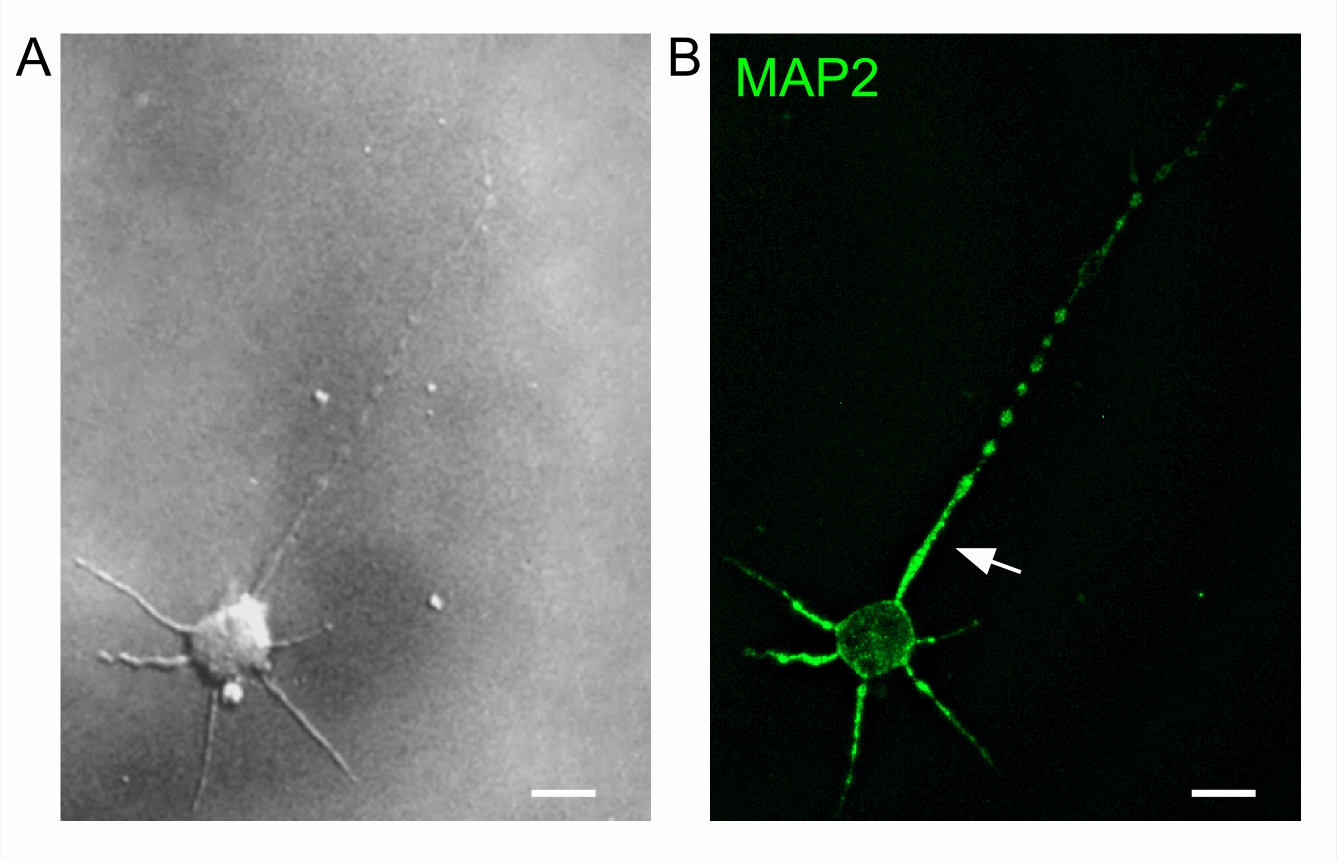


**Figure S3.** MAP2 is not selectively expressed in dendrites of hippocampal neurons after 3 days of cultures. (A) DIC and (B) fluorescence image of MAP2-expressing neuron. MAP2 is expressed in all neurites emerging from the soma, including the longest (indicated by the arrow) likely to be an axon. Scale bar, 10 μm.
