# Supplementary material for: Can Hippocampal Neurites and Growth Cones Climb over Obstacles?
Source: PLoS One. 2013 Sep 6;8(9):e73966. doi: 10.1371/journal.pone.0073966 (PMC3765352; doi:10.1371/journal.pone.0073966)
Supplement: Figure S4 — Hippocampal GCs and filopodia express Netrin-1 receptors DCC and UNC5A. Fluorescence confocal images of a hippocampal GC 24 h after plating stained for DCC (A) and UNC5A (B). (C) Merged images. White dotted line indicates the GC outline. Scale bar, 5 µm. (DOCX) [file pone.0073966.s004.docx]

**Supporting Information**


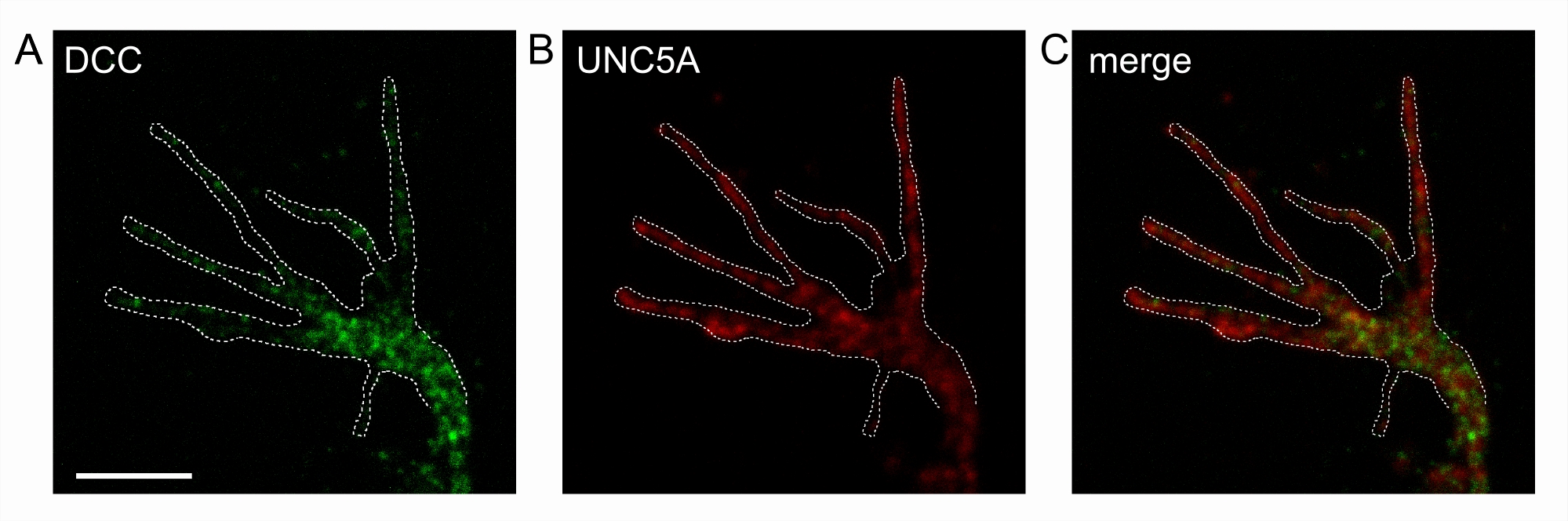


**Figure S4.** Hippocampal GCs and filopodia express Netrin-1 receptors DCC and UNC5A. Fluorescence confocal images of a hippocampal GC 24 h after plating stained for DCC (A) and UNC5A (B). (C) Merged images. White dotted line indicates the GC outline. Scale bar, 5 μm.
